# On the Application of Artificial Teeth upon the Atmospheric Pressure, or Suction Principle

**Published:** 1845-06

**Authors:** William Harnett

**Affiliations:** Dentist of New York.


### ARTICLE VII.

*On the Application of Artificial Teeth upon the Atmospheric Pressure, or Suction Principle.* By WILLIAM HARNETT, Dentist of New York.

HAVING, as I conceived, discovered something of great importance relative to the manufacture of suction plates, I hasten to forward to you, at the recommendation of various dentists of this city, the particulars for publication.

It is, as follows: After striking up the plate in the usual manner, engrave a border, or a line on the inner side of the plate, the gold must be rather thicker than is usual, as the engraving has to be cut at least half through the plate. After you have cut the border close to the edge of the plate, proceed to design, after the style of Louis XIV., the Renarssance, or any other elaborate mode, various scrolls, so that the whole of the inner surface of the plate may be covered, which will be sure to be the case, if the styles which I have named is adopted; as the various ramifications of the scrolls must, necessarily, be so minute, that no part can escape. Of course, the ground work of the scrolls must be cut away, and cut in deeply, and then *friezed*, as on this, principally, lies the advantage; as the friezing instrument (which is a tool used by *chasers*) has the power of levelling the ground-work, and, at the same time, making minute holes the size of a needle point thereon, thereby creating a vacuum which, coupled with the engraving, is, I consider, the greatest improvement that has yet been made in this branch of dentistry. When finished, the plate will have the same appearance as the back of a Geneva watch case; a locket, or any other piece of engraved jewelry. The advantage of this method over any of others that has yet been discovered, is, that it will sustain the plate better, and with greater firmness, and, likewise, that the food will not lodge in the plate, as it does in the old method, thereby creating a disagreeable effluvia. Again, it is as easy to cleanse as a plate that is quite plain, and the appearance, when well finished, has a most beautiful effect.

Should the above communication meet the approbation of my fellow professional brethren, my views will be fully answered.

